# From kinetics and cellular cooperations to cancer immunotherapies

**DOI:** 10.18632/oncotarget.8242

**Published:** 2016-03-21

**Authors:** Alain Trautmann

**Affiliations:** ^1^ INSERM, U1016, Institut Cochin, Paris, France; ^2^ CNRS, UMR8104, Paris, France; ^3^ Université Paris Descartes, Sorbonne Paris Cité, Paris, France; ^4^ Equipe Labellisée “Ligue contre le Cancer”, Paris, France

**Keywords:** immunotherapy, kinetics, cellular cooperation

## Abstract

In this review will be underlined two simple ideas of potential interest for the design of cancer immunotherapies. One concerns the importance of kinetics, with the key notion that a single cause may trigger two opposite effects with different kinetics. The importance of this phenomenon will be underlined in neurobiology, transcription networks and the immune system. The second idea is that efficient immune responses have been selected against pathogens, throughout evolution. They are never due to a single cell type, but always require multiple, complex cellular cooperations. One cannot recognize this fact and persist in the presently dominant T-cell centered view of cancer immunotherapies. Suggestions will be made to incorporate these simple ideas for improving these therapies.

## FROM KINETICS AND CELLULAR COOPERATIONS TO CANCER IMMUNOTHERAPIES

Twenty years ago, only a minority of immunologists and oncologists considered immunotherapy to be an effective strategy for cancer treatment. The situation has since radically changed, and several strategies are being used.

The anti-tumoral toxicity of antibodies targeting tumor [1] or vascular epitopes [2] is exerted via components of the immune system (NK cells, macrophages, or complement). Several of these antibodies have yielded clinical benefits, namely those used to treat blood cancers [3].

Other promising results have recently been obtained with antibodies directed against PD-1 and/or CTLA-4, i.e., T-cell surface molecules that exert inhibitory effects on T-cell responses, [4]. For instance, in a phase III clinical trial [5], 405 patients were treated either with an anti-PD-1 antibody or with investigator's choice of chemotherapy. Confirmed objective responses were reported in 32% of the patients treated with the anti-PD-1, versus 11% in the chemotherapy group. However, given the large percentage of patients displaying no response to treatment, real improvements to such approaches are required.

Antibody treatments activating T cells represented new variations of older trials designed to trigger antigen-specific T cells through different types of vaccination, including vaccination with antigen-loaded dendritic cells [6] or long peptides [7].

Another T cell-based cancer immunotherapy to attract attention recently is the adoptive transfer of patient T cells engineered to express CARs (*chimeric antigenic receptors*). These receptors are surrogate TCRs with a high affinity for a selected tumor antigen and boosted signaling activity. After their transfection and *in vitro* expansion, these cells are transferred to the patient. Impressive results have been obtained for a few B-cell malignancies refractory to all previous treatments. Thus, in a recent study [8], 14 patients with refractory chronic lymphocytic leukemia were repeatedly treated with CAR T cells targeting CD19, and complete or partial remissions were observed in 8 of them (57%). However, this approach has proved unsuccessful in the treatment of other blood cancers, and has never been shown to work for solid tumors [9], due to insufficient tumor infiltration and/or excessive nonspecific toxicity. Finally, general stimulation of the immune system with immunostimulatory cytokines, such as IL-2 (Royal 1996), IFNα [10], or GM-CSF [11], has also been tested, but with disappointing results and high levels of toxicity.

A major objection to these therapies is that they do not take into account two major features of efficient natural immune responses: their specific kinetic characteristics and the well documented cooperation between cells. Efficient immune system responses to infection are invariably based on complex cooperation between different cells rather than on the action of a single cell type. Strangely, the importance of this cooperation between cells is largely overlooked in oncoimmunology, where the prevailing view is highly T cell-centered.

Let us start with the lack of attention paid to kinetics in immunology, with few exceptions (see e.g. [12][13][14]). This may reflect the strong historical link with medicine, and with the medical custom of detailed classifications. This trend has been amplified by the power of flow cytometry, allowing the definition of an ever increasing number of cell subtypes. However, the time-dependence of the subpopulations is all too often ignored, with cell populations treated as if they were stable, which they are not.

The importance of kinetics is better taken into account in other biological fields, such as enzymology and neurobiology, probably thanks to a tradition of dialog with physicists and biophysicists. A brief detour through the field of neurobiology will help us to stress the importance of kinetics in analyzing functions. It will allow to present a general notion of the utmost importance: a single cause, or stimulus, may trigger opposite effects with different kinetics. We will use a few examples to illustrate and discuss this notion.

### Voltage-dependent Na^+^ channels, an archetypal example of a biological structure in which one stimulus triggers two opposite, successive effects

**Figure 1:**
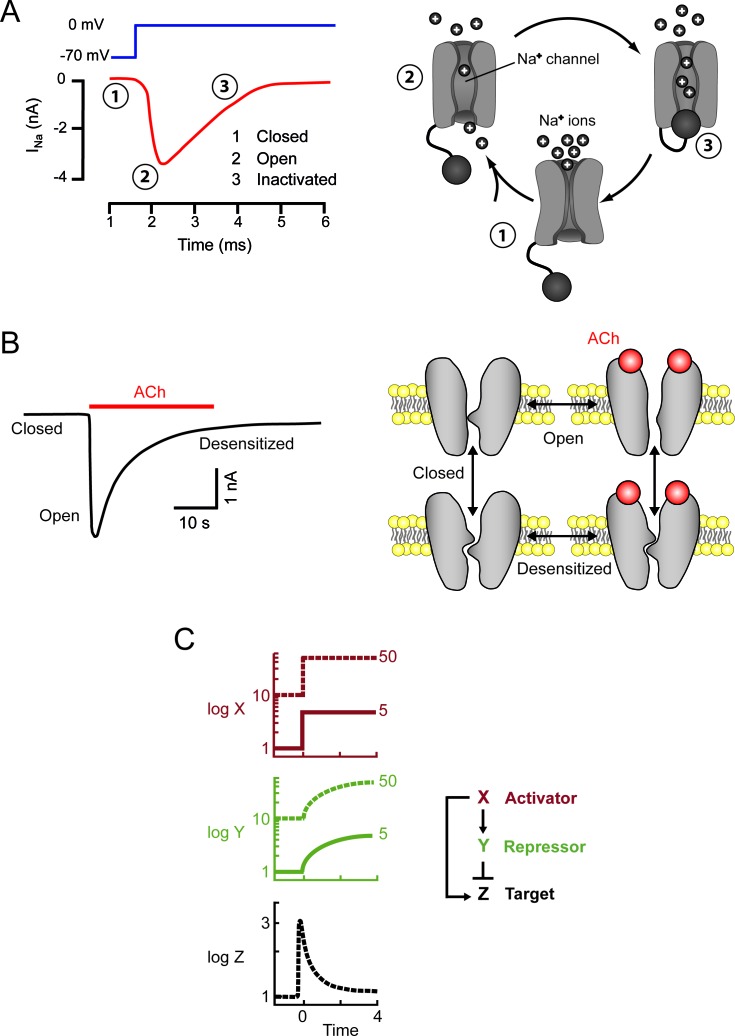
A single stimulus may elicit two opposite, successive responses Left: experimental data. Right: schematic models. **A.** In neurons, membrane depolarization (dark blue) elicits a transient inward current (red), due to the entry of Na^+^ ions into the cell. The channels, which are initially closed (1) first open (2) and are then inactivated/closed (3) by depolarization. The “ball and chain” model of inactivation is shown [51] **B.** Sustained ACh application triggers a transient inward current in the cell (muscle or neuron). Following ACh binding, nicotinic receptor channels first open and are then massively desensitized/closed. The desensitized conformation of AChR may exist in the presence and absence or bound ACh [52]. **C.** A transcription factor X immediately activates a target Z, and slowly activates a repressor Y, which then fully inhibits the target. In a such a system, activation of the target is transient (adapted from [15]). The model illustrates the fold-change paradigm: the same target response may be observed with stimuli of different intensities, the two outputs perfectly overlap.

Information travels along nerves in the form of action potentials. These action potentials are changes in the potential of the membrane, which locally loses the negativity of its resting state to become positive for one or a few milliseconds. Action potentials travel rapidly (1-100 m/s), reflecting the local propagation of a transient wave of Na^+^ channels opening. These channels are voltage-dependent and the initial membrane depolarization triggers two opposite consequences: the opening of the channel (activation), followed by its closure (inactivation) (Figure 1A). Only one effect is initially visible, but this effect is then fully masked by the second effect. This sequence translates a step stimulus into a response resembling the *derivative* of the stimulus: the Na^+^ channels open only immediately after the onset of the depolarizing stimulus. A key feature of this system is that the opening of some Na^+^ channels upon cell depolarization leads to the opening of other Na^+^ channels. This *positive feedback* underlies the kinetically explosive nature of action potential, which is brought to an end by inactivation.

### Desensitization of receptors to neurotransmitters and other ligands, a second example of a single cause triggering two opposite, successive effects

At the neuromuscular synapse, information is transmitted from a neuron to a muscle cell through the release of acetylcholine (ACh), which binds to receptors on the muscle, triggering two different events: opening of the nicotinic receptor-channel (AChR), followed by receptor desensitization, leading to channel closure. The second of these phenomena develops more slowly than the first, so we observe a transient opening of the AChR (Figure 1B). Initial activation on ligand binding, followed by receptor desensitization, is commonly observed not only with neurotransmitters, but also for chemokine receptors. This sequence of events explains the transient nature of most responses to neurotransmitters and chemokines. As shown in Fig 1, in these biphasic responses, one can distinguish *three states*: resting, activated, and desensitized/inactivated.

### Adaptation: a feature common to sensory systems, transcription networks and the immune system

The kinetic phenomena described above raise questions concerning adaptation and, thus, sensitivity to discontinuities. According to Weber's law, the smallest difference between two stimuli that can be discriminated by the sensory system is proportional to the magnitude of the stimulus. In other words, our sensory systems do not discriminate between absolute values; instead they detect fold-changes. When the visual system is stimulated with a constant level of light, it rapidly adapts such that the system detects only variations above this level, provided that these variations are large enough *relative to* this new level. In practice, variations of a few percent above the level of steady stimulation can be detected, whereas smaller variations cannot. This property underlies the ability of sensory systems to span huge variations of stimulus intensity.

Light may be detected over nine orders of magnitude : we can detect single photons in some conditions, and variations exceeding hundreds of millions of photons in others. This exceptional performance is dependent on a complex neuronal network and a combination of spatiotemporal mechanisms, including adaptation to background light levels.

This “fold-change detection” paradigm applies not only to sensory systems, but also to transcription networks. Theoretical backgrounds [15] have been used to demonstrate how an *incoherent feedforward loop*, in which an activator switches on both a gene and its repressor, can achieve this goal. If a single cause (the activator) triggers two effects, with activation slightly preceding full inhibition, then the transcriptional response can be considered to be the derivative of the stimulus. This model can be used to interpret the fold-detection paradigm (Figure 1C).

As discussed in more detail below, the immune system also adapts to chronic or constant stimulation. As a result, the immune system responds only to discontinuities [16][17], a feature of fundamental importance for its functioning. The sudden effraction of viruses or bacteria at the periphery of the organism (skin, mucosa) may be detected as discontinuities by a whole set of molecules present at the surface of cells from both the innate and adaptative immune systems. Thus, adaptation/sensitivity to discontinuities is a very general phenomenon, with features specific to each system. In particular, even though the immune system has to detect variations in its environment, it differs from sensory systems in one major aspect. The visual sensory system response to a single stimulus, light, which has a limited number of features (intensity, color, shape, velocity). The system is clearly structured along an axis extending from the photoreceptors to the brain. The immune system has no equivalent central structure. The detection system and information exchanges are distributed throughout the body and the system can detect thousands of different sorts of stimuli, which cannot be quantified in a simple manner. The fold-detection paradigm does not therefore apply to this system.

### Borders of the immune system

We now need to define the immune system. Any such definition is controversial by its very nature, because the immune system is nothing but a concept that has changed during the history of science. In the 1970s and 1980s, the most important feature of the immune system appeared to be its ability to make antibodies. The focus has since changed, and many immunologists now have a T cell-centered view of immunology. This focus will certainly change again in the future.

The immune system considerably evolved from primitive organisms, in which macrophages (Mφ) or their equivalent were the major cells involved in defense against pathogens, to vertebrates, which have many more types of immune cells, including antigen-specific T and B cells [18]. As our aim here is to find ways of improving cancer immunotherapy, we will focus on the mammalian immune system. Mφ pose a major problem in the definition of the mammalian immune system, because these cells are not only anti-infectious agents, they are also indispensable for the development of the organism [19]. They are required to eliminate dead cells, which are produced in large numbers in developing organisms, and to control the development of the stroma which envelops the organs in normal conditions or after a lesion. They remain essential for the maintenance of adult tissues.

The definition of the immune system is further complicated by the abundant commensal flora present in the intestine. This has led some authors to consider the superorganism formed by the organism and its flora, and to suggest that the main function of the immune system is to maintain the homeostasis of this superorganism [20]. Such a broad definition of a homeostatic immune system may be considered too vague to be particularly useful, especially when considering evolutionary pressures. A definition that is more helpful as a conceptual tool is that the *mammalian immune system* consists of a *set of different cells* (B and T lymphocytes, NK cells, neutrophils, Mφ, dendritic cells) *and of the molecules they secrete* that may act collectively to exert efficient anti-infectious effects. Consistent with this definition is the recognition that pressures on the immune system during the course of evolution have optimized the efficacy of these anti-infectious effects. As discussed below, the immune response always includes a reactive, acute phase followed by a return of the immune system to an equilibrium.

The immune system may therefore be seen as a major, but not exclusive, *subset of a larger anti-infectious device* that also makes use of mechanisms present in all cell types. Almost all cells can detect the presence of viral double–stranded RNA and secrete IFNα/β upon viral infection. Similarly, upon bacterial infection, any cell type (including, in particular, mucosal epithelial cells) can detect the presence of the bacteria and trigger NF-κB-dependent IL-8 secretion, thereby alerting neutrophils [21]. Such ubiquitous triggering mechanisms play a key role in the anti-infectious device, but are not specific to the immune system. More precisely, IFNα/β can initially be produced, in small quantities, by any cell type. However, the immune system contains a single cell type (plasmacytoid dendritic cells) able to produce this compound in huge amounts [22]. IFNα/β may therefore be considered to be both out and in the immune system.

With our definition of the immune system (a set of cells and the molecules they secrete to fight infection), Mφ are both *part of and outside the immune system*. Conceptually, they lie at the borders of the immune system and, physically, they are frequently particularly abundant at the periphery of organs.

Key phenomena take place at these blurred borders, particularly in the intestine, the largest interface between the interior and exterior of the body. There is an extraordinary concentration of bacteria at this border, with which the mammalian host must establish a well-controlled dialog. Immune cells are highly abundant at this interface, at which the immune system encounters some of its trickiest challenges: active defense against pathogens without unnecessary overreaction that might damage normal tissue or the associated commensal organisms essential for both food assimilation and for preventing the development of other toxic bacteria. Indeed, the presence and abundance of non-toxic intestinal bacteria contribute to the defense of the body against potentially toxic bacteria [23]. Like the immune system, commensals constitute another *subset of a larger anti-infectious device.*

A key constraint acting on the immune system is that it must not trigger autoimmune attack. This constraint has led to the selection of processes to prevent damage to *self*. An economical definition of the self is *what is permanent* in an organism or at least what changes only slowly. However, *self* is not a notion easy to define. In particular, *commensals* could be viewed both as self and as non-self. They are part of self because they are *permanently* present in the intestine, in very large numbers, and play a key role in our homeostasis. But they can also be seen as non-self because we are not born with them, they can have hosts other than humans and the evolutionary pressures acting on them are different from those acting on humans. We can therefore consider them to be part of a self with blurred borders.

The notion of self/non-self is irrelevant at the level of single cells. T cells are not activated by non-self ligands, but simply by a sudden change in the amount of a recognized ligand. The concept of self becomes important and useful only as an *emergent* property of a multicellular structure, the organism, which includes permanent, or at least slowly changing, components.

### Importance of kinetics in immunology, as illustrated by the case of viral infections

Viral infection rapidly triggers a sequence of events. As described above, virus detection leads first to a non-immune response: any cell infected with a virus produces IFNβ and IFNα, and shuts down its own translation processes and those in neighboring cells. Sentinel cells (resident Mφ and innate lymphoid cells) raise the alert, triggering the recruitment of inflammatory monocytes/Mφ, thereby initiating the immune response. By rapidly ingesting infected cells, Mφ make an immediate contribution to viral clearance. Together with dendritic cells (DC), they also recruit T cells, and present them viral antigens, leading to the initiation, several days later, of a T-cell response. Only then do the CD8 T cells become key antiviral actors. Beforehand, they have an absolute requirement for the initial help of Mφ, DC, and CD4 T cells, both for their initial recruitment and for their subsequent activation. CD4 T cells also play a key role in the development of antigen-specific B cells, which differentiate into plasmocytes and produce antibodies (some of which are, hopefully, neutralizing). The rare CD8 T-cell clones able to recognize viral antigens at the surface of DC then proliferate very rapidly. A detailed study of the kinetics of this response in individuals vaccinated against yellow fever [24] showed that specific anti-viral T cells took several days to reach large numbers, with a peak about two weeks after infection. If these T cells kill infected cells efficiently enough then the spread of the virus is halted.

Even if it takes days and must be preceded by an innate response, the development of the T-cell response is accelerated by *positive feedback mechanisms*. The clonal exponential expansion of virus-specific T cells is characterized by a constant acceleration, as in a positive feed-back system. The proliferating T cells then produce a growth factor, IL-2, and express CD25, the high-affinity receptor for IL-2. This triggers an autocrine and paracrine feedback loop for T-cell expansion and differentiation.

The expansion of the virus-specific T-cell population is followed by a phase of contraction. This population rapidly decreases in size, through a series of mechanisms, the first of which is the fratricide phenomenon of FasL-dependent AICD (*activation-induced cell death*). Other T cells die by starvation. They require the growth/survival factor IL-2 if they are to stay alive, but *IL-2 production by T cells is only transient*, peaking *in vivo*, about six days after the recently activated naive murine T cells begin to respond (this time lag is only two days for memory T cells) [25]. Similarly, *CD25 is expressed only transiently* on activated T cells. Its expression peaks one day after the initiation of the response, and it disappears within one week [26]. With insufficient CD25 and IL-2, the activated T cells die. As IL-2 is consumed by T cells, an overpopulation of T cells results in a shortage of available IL-2. This IL-2-dependent control of the size of the T cell population is further tuned by specialized T cells which have a strong impact on the available IL-2. Thus, Th2 CD4 T cells act as IL-2 producers, a production that may benefit T cells that are not producing IL-2. Amongst the pure IL-2 consumers, regulatory T cells completely depend upon other T cells for the IL-2 that they consume. When their number is large, they act as a sink for IL-2 and thus accelerate the shrinkage of the global IL-2-dependent T cell population.

In addition to the mechanisms contributing to the collapse of the recently expanded T-cell population, there are mechanisms decreasing the functional efficiency of the remaining T cells, due to the expression of inhibitory receptors, such as PD-1 and CTLA-4 [27]. As PD-1 expression begins one day after the onset of CD25 expression, it should be considered *a marker of activated T cells*, not only of inactivated or desensitized cells. An understanding of the kinetics of expression of molecules such as CD25, IL-2, PD-1 is essential if we are to comprehend the functional state of these cells.

After the contraction phase, the immune system does not return to its initial state, because a new, small population of long-lived memory T and B cells (antiviral in this case) has appeared, which will ensure a faster response to subsequent viral attacks. We have considered the case in which the contraction of the T-cell population follows similar kinetics to the disappearance of the virus. However, if the response is not efficient and the virus persists, the contraction phase and the generation of inhibitory receptors nevertheless occurs. It is not the elimination of the virus that stops the immune response. The expansion-contraction sequence is triggered right at the start of the infection. This is another example of the principle that a single cause may induce two opposite and successive effects. If the virus persists, the T cells remaining after the contraction phase are not memory T cells but dysfunctional T cells (sometimes called anergic, exhausted or desensitized). This state is not definitive. It can be reversed by the removal of chronic stimulation, when cells are maintained in culture for example [28].

These desensitizing mechanisms should not be viewed as a defect or problematic limitation of the efficacy of the T cell-mediated antiviral response. In the absence of such mechanisms, the overactivated immune system would cause off-target attacks on normal tissues, resulting in catastrophic autoimmunity. Indeed, despite these mechanisms, a number of autoimmune diseases are known to be triggered by viral infection [29]. It may seem strange that autoimmune diseases are often chronic when efficient antiviral responses are necessarily transient. One reason is that even though immune activity is not very efficient in chronic autoimmune diseases, its destructive consequences accumulate slowly and may be irreversible. Furthermore, autoimmune diseases are often not truly chronic, instead resulting from repeated transient attacks. Finally, autoimmune diseases are usually associated with local chronic inflammation, a process in which non immune cells, i.e., activated fibroblasts [30], play a major role.

Thus, multiple mechanisms contribute to the fact that T cell-mediated immune responses to a viral attack are usually transient.

### The absence of an efficient antitumoral immune response is predictable

**Figure 2:**
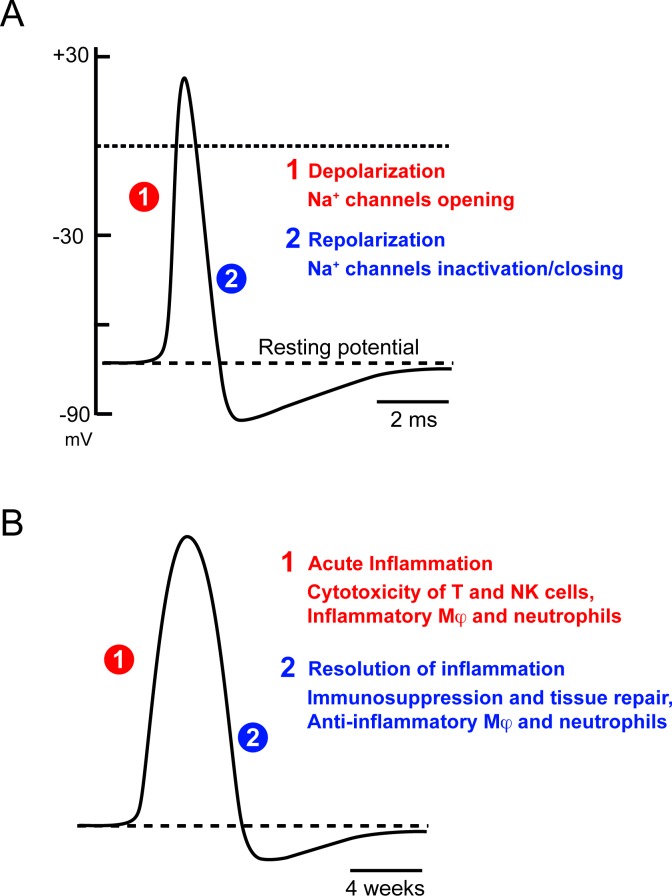
Transient nature of biological responses **A.** Action potential **B.** Immune response. Adapted from [47]. Similar times courses with very different time scales.

Let us summarize some of the conclusions reached so far. We have seen that, in many biological systems, a single cause can trigger two opposite and successive responses, with activation preceding marked inhibition. Such phenomena are general, applying to neuronal Na^+^ channels, neurotransmitters and chemokine receptors, transcription networks, and the immune system. Figure 2 illustrates the striking similarity in the time course of action potentials and immune responses, over different time scales.

The functional consequences of this phenomenon are entirely system-dependent. In the immune system the specific features of these transient responses are as follows. First, as positive feedback mechanisms play an important role in accelerating and strengthening the initial response, inhibitory mechanisms are subsequently required to prevent an explosive, uncontrolled response. Second, the immune system, which is highly effective against pathogens, is very powerful, and this power poses a constant threat of bystander damage to self. A key feature of self being its permanence, an absence of response to sustained signals is required to avoid damage to what is permanent, to prevent autoimmunity. This may explain why repetitive stimulations of T cells, B cells or NK cells are integrated in chronic stimulations leading to anergy/desensitization.

In humans, tumor development is thought to start very slowly, with an initial growth phase lasting for several years followed by a secondary acceleration in which all the controls have been removed and tumor growth becomes noticeable. A normal stromal environment limits tumor development [31]. At some time point, under the influence of tumor cells, the stroma may become activated and contractile, with serious consequences. Indeed, tumor growth requires not only multiple mutations in the tumor cells themselves, but also changes in their interactions with neighboring cells [32]. There may therefore be a *switch in the function of the tumor-stroma border*, from a tumor-controlling to a tumor-promoting role. The immune system also exerts an additional control, particularly for metastasis development, which is facilitated in the absence of CD8 T cells [33].

Thus, both normal stroma and immune cells may slow tumor development. With such slow kinetics (absence of discontinuity), an intense immune response cannot be triggered. If a weak, inefficient immune response is nevertheless transiently triggered, then the specific anti-tumor cells become desensitized, because the tumor has become a permanent component of the organism, at the time scale of a lymphocyte (a few weeks or months). Even though the immune system is one of the systems limiting the occurrence of some cancers, the frequent *lack of efficiency of anti-tumoral immune responses* should not be viewed as a defect of the of the immune system, but an expected, *normal feature* of this system.

### From anti-infectious to anti-tumoral immune responses

When comparing anti-infectious and anti-tumoral immune responses, a key question arises concerning the interactions between T cells and Mφ. The T cell-centered view of cancer immunotherapy has recently been reinforced by the striking clinical trials mentioned at the beginning of this paper, with antibodies against PD-1 or CTLA-4 on the one hand, and T-cell CARs on the other. The spectacular results of these trials have bolstered the existing dogma: *The T cells are the good guys* against the tumor, we must simply give them more weapons to allow them to strike more forcefully. It's just a question of intensity.

The other side of the coin is that *Mφ are often considered to be the bad guys*: in many cases, their abundance in the tumor is correlated with a poor prognosis. The initiation and development of many cancers have been associated with chronic inflammation. Based on this observation, the prevailing view is that inflammation and inflammation-associated cells, such as Mφ, exert protumoral effects.

Inflammation, like many other biological phenomena, is normally transient. It may have many possible origins — internal or external, microbial, physical or due to toxins — but it generally presents with a rapid onset followed by a phase of resolution [34]. Evolution has selected this acute, controlled inflammatory process to optimize the defense of the organism. The problem is that the resolution phase does not include the full adaptation/desensitization/anergy described above for other biological phenomena. As a result, *chronic stimulation may lead to chronic inflammation*, which may even become self-sustained after the cessation of a prolonged stimulus, because fibroblasts may have locally switched locally and permanently to an activated stated [30]. Chronic inflammation should not be confused with basal inflammatory tone, which is linked to the presence of the commensal flora [35] and keeps the immune system on alert without actually triggering a real, acute response, with positive and negative feedback. Mφ can contribute to both acute and chronic types of inflammation. In addition, independently of inflammation, they contribute to the development and maintenance of all tissues, including tumors. These characteristics have led to Mφ being seen as the bad guys by most oncoimmunologists (for a widely cited review, see [36]).

Ten years ago, Mφ were generally considered to be of two types, with M1 Mφ associated with inflammation, as opposed to M2 Mφ, which were considered responsible for anti-inflammatory responses [37]. Overall, the inflammation-Mφ association was considered to be pro-tumoral, particularly in the presence of B cells and antibodies contributing to this inflammation [38]. M1 and M2 have since been recognized as the extremes in a spectrum of phenotypes, with the Mφ present in a tissue usually presenting mixed M1 and M2 features: the binary distinction is too simplistic [39]. In addition, several groups have shown that Mφ plasticity can be exploited for anti-tumoral purposes, through activation elicited by IFNγ, TLR agonists, and anti-CD40 antibodies (reviewed in [40]). However, this remains a minority view, with the prevailing dogma remaining that immunotherapy for cancer will be improved by the elimination of tumor-associated Mφ.

Making use of the anti-infectious immune response to fight cancer is an old idea that still deserves to be defended today. This was already the conviction of William Coley, at the end of the 19th century. Coley noticed a striking coincidence between major infections and subsequent “spontaneous” tumor regression [41]. For reasons that remain unclear, such regressions wer more frequently observed for sarcoma (of mesenchymal origin) than for carcinoma (of epithelial origin) [42]. Coley exploited this finding throughout his career, but very little research is carried out in this area today.

One of the very few clinical applications arising from Coley's work is the intravesical injection of BCG (*Bacillus Calmette-Guérin*) for the treatment of bladder cancer. This approach works by triggering an intense immune response, beginning with massive neutrophil infiltration [43]. All attempts to extend this result to other cancers have failed, probably because the bladder is in the unusual position of being located within the body, but directly connected to the exterior. This specific location, at the border between the interior and exterior, makes it possible to introduce a very high concentration of attenuated but living bacteria, triggering an intense local immune reaction. The introduction of a similar number of bacteria directly into an irrigated organ would cause extensive damage and lead to dissemination of the bacteria throughout the body.

Oncolytic viruses can also be used to exploit anti-infectious responses to fight cancer. These viruses present a marked tropism for tumor cells, in which they replicate more efficiently than in normal cells. Oncolytic viruses were initially used in the hope that they would have a direct oncolytic effect, killing tumor cells due to their massive replication. However, it then became clear that the essential anti-tumoral effects of these viruses were not direct. They were instead due to the immune response triggered by their presence (for a review, see [44]). However, treatments with oncolytic viruses have present limits, which need to be analyzed. These viruses often have a modest anti-tumor effect because they fail to spread properly within the tumor. Furthermore, the efficacy of the *immune* system against viruses is such that the virus-induced response is rapidly extinguished. For instance, in a clinical trial performed with adenoviruses expressing melanoma-associated antigens, the results were disappointing, possibly because anti-viral antibodies were present before vaccination, blunting its effects [45]. Nevertheless, the first oncolytic virus therapy for the treatment of melanoma lesions had been FDA-approved in october 2015 (http://www.fda.gov/NewsEvents/Newsroom/PressAnnouncements/ucm469571.html).

In addition to the use of controlled pathogens, we could design artificial ways of *mimicking* a local viral attack, for tumors in appropriate locations, with the goal of simultaneously activating multiple actors of the immune system. We have recently applied this strategy in an implanted tumor model in mice. We used a compound vaccine aimed to trigger simultaneous Mφ and T-cell responses [46]. The vaccine, injected into the tissues adjoining the tumor, included IFNα to trigger an anti-viral response, and a tumor antigen targeted to DC through its chimerization with a Shiga toxin. In this model, tumor regression was observed in nearly all cases, whereas the two components of the vaccine were almost totally ineffective when used in isolation. We obtained several lines of evidence suggesting that both Mφ and T cells were required for this effect. Their interactions were complex, and each of these cell types can be seen as acting both upstream and downstream of the other. Following vaccination, the Mφ arrive first and are required for the subsequent recruitment of large numbers of T cells, as in the anti-infectious response. Conversely, the few T cells arriving with the Mφ are required for Mφ activation. However, after a few days of strong tumor regression, some of the tumors begin to grow again. This should be seen as a normal outcome, corresponding to the usual phase of immune response inactivation. We are now analyzing the principal mechanisms at work during this secondary growth, and ways of inhibiting them at appropriate time points.

### What next?

Obviously, it is easier to treat implanted tumors in mice than to treat oncogene-induced murine tumors, and the extension of these results from mice to men will constitute another major hurdle. However, a few simple principles proposed a few years ago [47], further developed in this paper, should make it possible to improve immunotherapies for cancer. These principles can be summarized as follows.

First, efficient immune responses are those that have been *selected on the basis of their efficacy against pathogens*, during the course of evolution. They are never mediated by a single type of cells, instead systematically requiring complex cooperation between multiple cell types. When applying this notion to cancer immunotherapy, it may be helpful to think in terms of *dynamic cellular networks*. On the one hand, there is the tumor ecosystem, including tumor and stromal cells; on the other, the immune infiltrate. If we wish to destabilize the tumor ecosystem, it is highly desirable to attack a number of different points simultaneously and to stimulate the immune infiltrate at several levels.

Second, efficient immune responses are usually of a *blitzkrieg* type. At the scale of an organism, anti-infectious responses last a few days, if there is no uncontrolled complication, and are always followed by inactivation phases that must be dealt with. The transient nature of immune responses is necessary to avoid autoimmunity.

The question of kinetics is also important for the analysis of intra-tumoral immune infiltrates. Such analyses, performed in resected tumors, are widely used for prognosis purposes. One should keep in mind that the detailed nature of an immune infiltrate in a growing tumor provides a snapshot of an immune response that has failed to control the tumor. It does not include any information about the kinetics of the response. It can provide elements related to the initial spontaneous response, but it yields no insight into the nature of an efficient anti-tumoral response. This highlights the importance, whenever possible, of *analyzing in detail the kinetics of efficient anti-immune responses* associated with tumor regression [48].

Here are a couple of examples of how these notions could be better used in combined therapies. The first example concerns therapies based on *CAR T cells*. As explained above, one of the reasons for the failure of these treatments against solid tumors is the lack of specificity of these T cells, resulting in off-target effects and excessive toxicity [49]. This problem is mostly treated with corticosteroids or other anti-inflammatory molecules. However, simply limiting the intensity of the attack cannot improve its specificity. It is not surprising that attempts to mount an immune response based entirely on T cells, without aiming at stimulating other partners, have to involve a violent T-cell response, which may turn out to be poorly controlled. The risk is further increased by attempts to use CAR T cells in which PD-1 and CTLA-4 have been knocked out. Why not aim to achieve cooperation between *local inflammation at the level of the tumor* and smaller numbers of CAR T cells? Local inflammation can be achieved with oncolytic viruses or by the peritumoral injection of IFNα. Such local signals might favor the recruitment of circulating activated T cells, as in our murine model [48]. We have observed that efficiency of local inflammation elicited by IFNα was much larger than that elicited by systemic IFNα. A comparison with anti-infectious responses helps to understand that the induction of *local inflammation* is more likely to be successful than the systemic use of IFNα more commonly used. The logic at work in such a proposal is diametrically opposed to that underlying the combination of a large dose of CAR T cells with corticosteroids.

Second example: despite the spectacular results achieved with *anti-PD-1 and anti-CTLA-4 therapies*, these approaches do not take into account the transient nature of efficient immune responses. Anti-PD-1 and anti-CTLA-4 antibodies aim at reducing inhibitory mechanisms in T cells, limiting the strength of the inactivation phase, but they ignore the activation phase. Such treatments would be greatly improved by the introduction of a prior phase of deliberately triggered activation, mimicking a local response to infection, for example. An effective way of achieving this end would be to combine the action of oncolytic viruses with an anti-PD-1 approach [50].

In conclusion, if there is no magic cancer immunotherapy, a few basic but overlooked principles could help the design of more efficient immunotherapies. They are summarized a last time.

Anti-infectious immune responses are based on cell-cell cooperations. Given their high efficacy, they should be used as templates for anti-tumoral responses, and include multiple simultaneous stimuli.

Efficient anti-infectious immune responses are biphasic, and the importance of such kinetics must be taken into account.

Examples of applications of these principles have been presented above. Such notions are at odds with the principles often applied in clinical trials, which usually start with a dose escalation, to adjust the intensity of the blow judged in terms of efficacy/toxicity ratio, but which pay much less attention to the context in which this blow is delivered. In this perspective, we hope that the reasoning outlined here will be of value, and will encourage different, hopefully successful approaches.
